# Development of patient-specific osteosynthesis including 3D-printed drilling guides for medial tibial plateau fracture surgery

**DOI:** 10.1007/s00068-023-02313-w

**Published:** 2023-06-30

**Authors:** Nick Assink, Miriam G. E. Oldhoff, Kaj ten Duis, Joep Kraeima, Job N. Doornberg, Max J. H. Witjes, Jean-Paul P. M. de Vries, Anne M. L. Meesters, Frank F. A. IJpma

**Affiliations:** 1 Department of Trauma Surgery, University of Groningen, University Medical Center Groningen, HPC BA13, Hanzeplein 1, 9713 GZ Groningen, The Netherlands; 2 3D lab, University of Groningen, University Medical Center Groningen, Groningen, The Netherlands; 3 Department of Surgery, University Medical Center Groningen, Groningen, The Netherlands

**Keywords:** Tibial plateau fracture, Medial tibial plateau, Three-dimensional, 3D printing, Virtual surgical planning, Guided surgery, Patient-specific implant, PSI

## Abstract

**Purpose:**

A substantial proportion of conventional tibial plateau plates have a poor fit, which may result in suboptimal fracture reduction due to applied -uncontrolled- compression on the bone. This study aimed to assess whether patient-specific osteosyntheses could facilitate proper fracture reduction in medial tibial plateau fractures.

**Methods:**

In three Thiel embalmed human cadavers, a total of six tibial plateau fractures (three Schatzker 4, and three Schatzker 6) were created and CT scans were made. A 3D surgical plan was created and a patient-specific implant was designed and fabricated for each fracture. Drilling guides that fitted on top of the customized plates were designed and 3D printed in order to assist the surgeon in positioning the plate and steering the screws in the preplanned direction. After surgery, a postoperative CT scan was obtained and outcome was compared with the preoperative planning in terms of articular reduction, plate positioning, and screw direction.

**Results:**

A total of six patient-specific implants including 41 screws were used to operate six tibial plateau fractures. Three fractures were treated with single plating, and three fractures with dual plating. The median intra-articular gap was reduced from 6.0 (IQR 4.5–9.5) to 0.9 mm (IQR 0.2–1.4), whereas the median step-off was reduced from 4.8 (IQR 4.1–5.3) to 1.3 mm (IQR 0.9–1.5). The median Euclidean distance between the centre of gravity of the planned and actual implant was 3.0 mm (IQR: 2.8–3.7). The lengths of the screws were according to the predetermined plan. None of the screws led to screw penetration. The median difference between the planned and actual screw direction was 3.3° (IQR: 2.5–5.1).

**Conclusion:**

This feasibility study described the development and implementation of a patient-specific workflow for medial tibial plateau fracture surgery that facilitates proper fracture reduction, tibial alignment and accurately placed screws by using custom-made osteosynthesis plates with drilling guides.

## Introduction

Fractures of the tibial plateau are usually composed of complex fracture patterns including multiple bone fragments. During surgical treatment of these fractures, the main goals are to re-establish joint stability, achieve normal limb alignment and restore the articular surface [1, 2]. Surgical treatment of intra-articular tibial plateau fractures consists of closed or open reduction and internal fixation using screw or plate fixation. Adequate fit of the plates is of great importance for both the biomechanical stability and to minimize soft-tissue irritation [3–5]. In addition, plates who match the anatomical shape of the bone can serve as a template which facilitates indirect fracture reduction. In the last decades, plate osteosynthesis has continuously evolved into the present generation of off-the-shelf locking plates which match the average shape of the tibia [6]. However, both clinical experience and literature suggest that in a substantial proportion of the population these plates still have an improper fit [5]. The recent studies regarding statistical shape models of the tibia confirm this assumption by showing large anatomical bone variations, especially around the tibial plateau and across ethnic groups [4, 7].

**Fig. 1:**
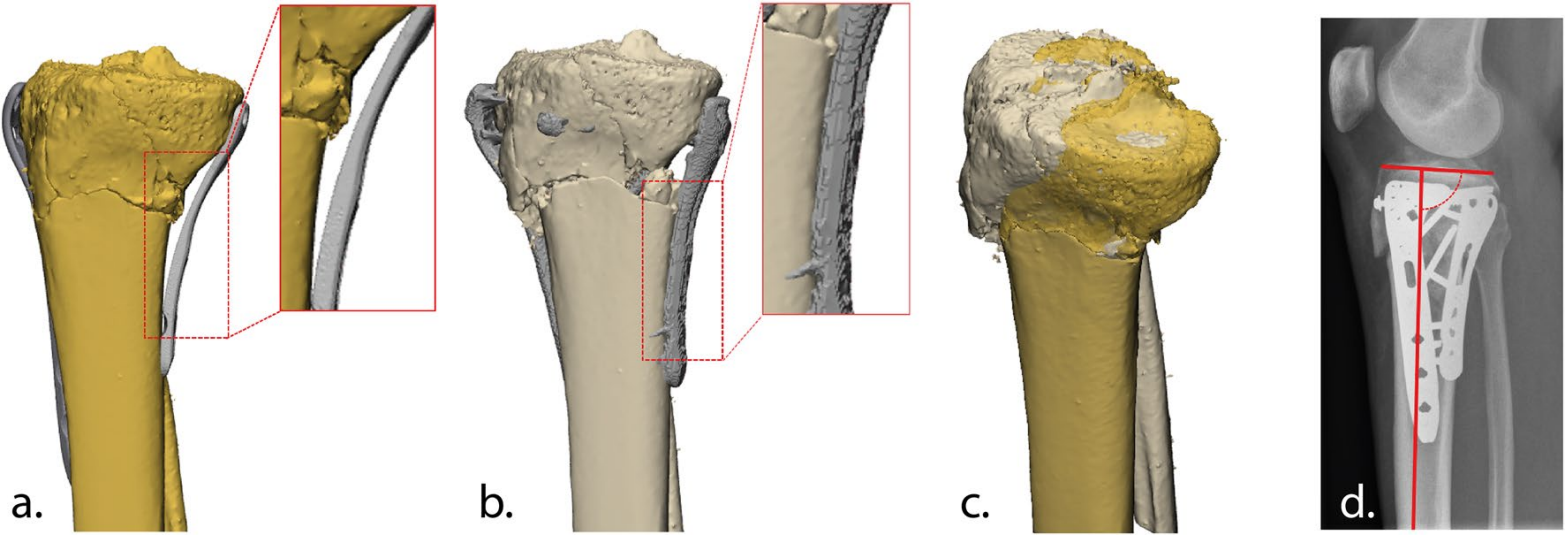
Clinical case of a female patient in her 40 s, who was treated for a Schatzker 6 tibial plateau fracture. **a** Surgical plan involving dual-plating including a posteromedial plate which had substantial space in between plate and bone due to suboptimal fitting. **b** Postoperative result: Due to uncontrolled compression on the bone with the poor-fitting posteromedial plate, the tibial shaft and the fragments are not properly aligned. **c** Planning (yellow) vs. postoperative result (grey). **d** Postoperative result on a lateral radiograph shows reduced sagittal alignment (2°) (colour figure online)

A plate with a poor fit may result in suboptimal fracture reduction and therefore inadequate tibial alignment due to applied -uncontrolled- compression on the bone during surgery, which may result in residual displacement of fracture fragments. One of our recent clinical cases clearly illustrates the potential negative effect of a poor fitting medial tibial plateau plate. This patient was treated for a Schatzker 6 tibial plateau fracture with dual plate fixation. Compression on the bone with a poor-fitting posteromedial plate led to inadequate sagittal alignment of the tibia (Fig. 1). Medial tibial plateau plates in particular often do not fit properly in our experience, which could be explained by the variation in size and slope and this is slightly more prominent at the medial plateau when compared with the lateral plateau [4]. This case illustrates that despite the progress in osteosynthesis plates and surgical techniques, even experienced surgeons do not always achieve adequate articular reduction and tibial alignment. Similar clinical experiences were confirmed by Meulenkamp et al., who reported that in 30% of the surgically treated tibial plateau fractures, an unsatisfactory reduction of fracture fragments was achieved [8]. Achieving adequate fracture reconstruction is essential since it is associated with improved functional outcome and reduced risk of progressive osteoarthrosis and decreased risk on conversion to a total knee arthroplasty (TKA). [9, 10]

Recently, we developed an innovative surgical procedure for fracture treatment using 3D virtual surgical planning and custom-made patient-specific osteosynthesis plates with drilling guides [11, 12]. Two previous studies showed that a personalized approach with perfect implant fitting facilitates proper fracture reduction and yielded good clinical outcomes. We hypothesized that this new patient-specific approach would benefit tibial plateau fracture treatment and may result in optimal osteosynthesis plate fitting, templating for adequate fracture reductions, and accurate screw placements. The clinical relevance of our study is the currently available medial tibial plateau plates often do not fit properly and hamper fracture reduction. A personalized approach might overcome this issue. The aim of this study is to assess whether our innovative workflow can be used to fabricate patient-specific implants for medial tibial plateau fracture surgery. We assessed the feasibility, accuracy, and efficiency of this innovative procedure through six cadaveric knees.

## Materials and methods

### Specimens and fracture fabrication

Three full body Thiel embalmed human cadavers were obtained from the anatomy department [13]. Three pure medial articular fractures (Schatzker 4, one fragment) and three multifragmentary complete articular fractures (Schatzker 6, three fragments) were created by a consultant trauma surgeon. The fractures were made in each knee using an oscillating saw and an osteotome through a parapatellar approach. The fabricated fractures and the degree of initial displacement were comparable with fractures seen in clinical practice [14]. A CT scan of the lower extremities was made of each cadaver according to our standard imaging procol used in clinical practice (0.6 mm slice thickness, voxel size 0.4 mm), which is the starting point for our 3D surgical planning.

### 3D surgical planning

3D models of all cadaveric knees were created using the Mimics Medical software package (Version 22.0, Materialise, Leuven, Belgium). The CT data (DICOM files, Digital Imaging and Communications in Medicine) was imported after which a segmentation process was performed by using a preset bone threshold (Hounsfield Units ≥ 226). All bones in the knees were separated to individual masks, by combining both region growing and split mask functions. This process was repeated in order to separate the independent fragments. Subsequently the fragments were checked and if needed manually separated from adjacent fragments. Based on a template of a healthy tibia, the fracture was virtually reduced by repositioning the fragments to their anatomical location, after which the 3D models were imported into the 3-matic software (Version 15.0, Materialise, Leuven, Belgium). The optimal screw trajectories and lengths were determined taken into account the fracture pattern. Based on these screw positions, a patient-specific plate was designed in a multidisciplinary meeting with surgeons, technical physicians, and engineers.

The patient-specific titanium plates for the tibia were designed according to our well-established workflow for the manufacturing of patient-specific plates [11, 12]. The shape of the plate was designed to perfectly deliver the preferred screw locations and directions. The customized titanium osteosynthesis plates were created using 3-Matic software version 15.0 (Materialise), Solidworks Professional software version 2020 (Dassault Systèmes Solidworks), and the Geomagic package for Solidworks (3D Systems). The plates were made of a medical grade titanium alloy by CNC miling using a 5-axis milling machine. Fabrication was done by a regional ISO 13485 certified medical company (Witec Medical B.V., Stadskanaal, The Netherlands).

**Fig. 2:**
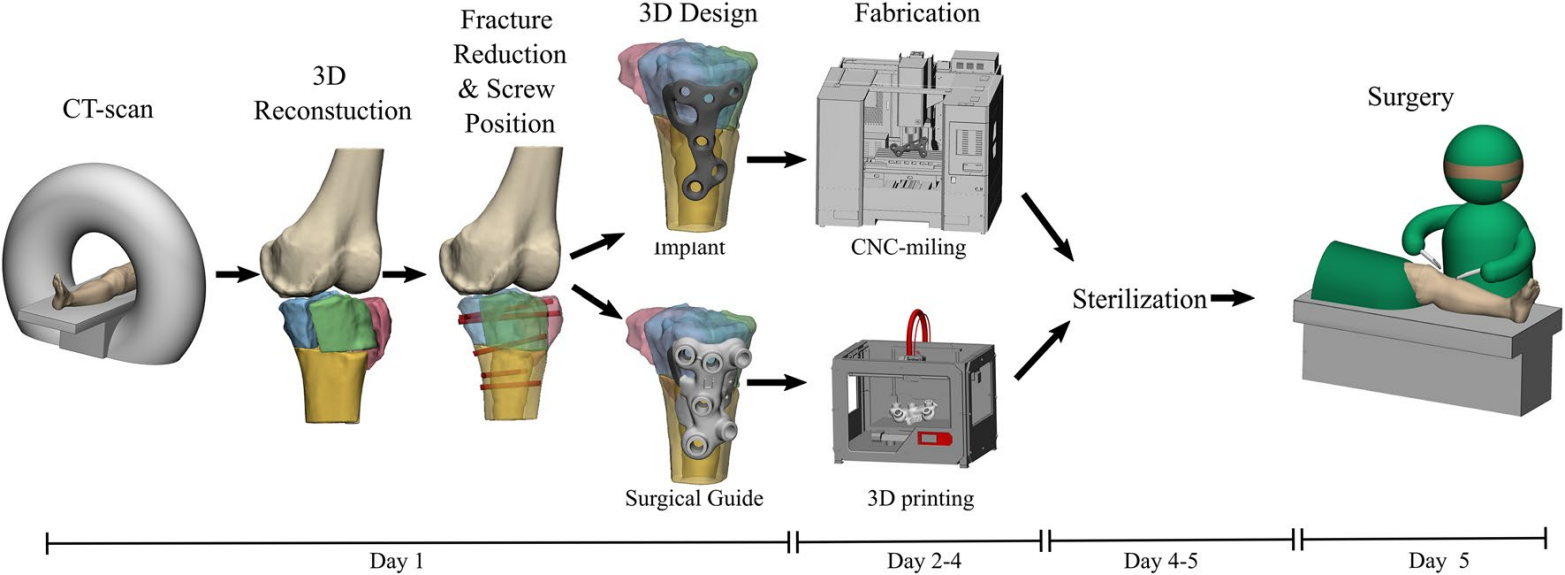
Workflow of manufacturing the patient-specific implant and the accompanied surgical guides for management of medial tibial plateau fractures. The whole workflow of designing, producing, sterilizing and (clinical) application is feasible within days in our clinic

The drilling guides, which were designed to fit on top of the customized plates, assisted the surgeon to position the plate and steer the screws in the preplanned direction. The drilling guides consisted of multiple cylinderic holes in which a stainless-steel drill sleeve (316 L, 25 mm in length, with an inner diameter of 2.9 mm for a 2.8 mm drill) could be inserted to guide the drill. In addition, bone supporting extensions were added to the design, which directed the plate to its intended position. After the designing process, the guides were 3D-printed by selective laser sintering using polyamide 12 (PA12), which can be sterilized for usage during the operation. The entire 3D surgical planning workflow from CT scan to surgery is depicted in Fig. 2.

### Patient-specific osteosynthesis plate design

**Fig. 3:**
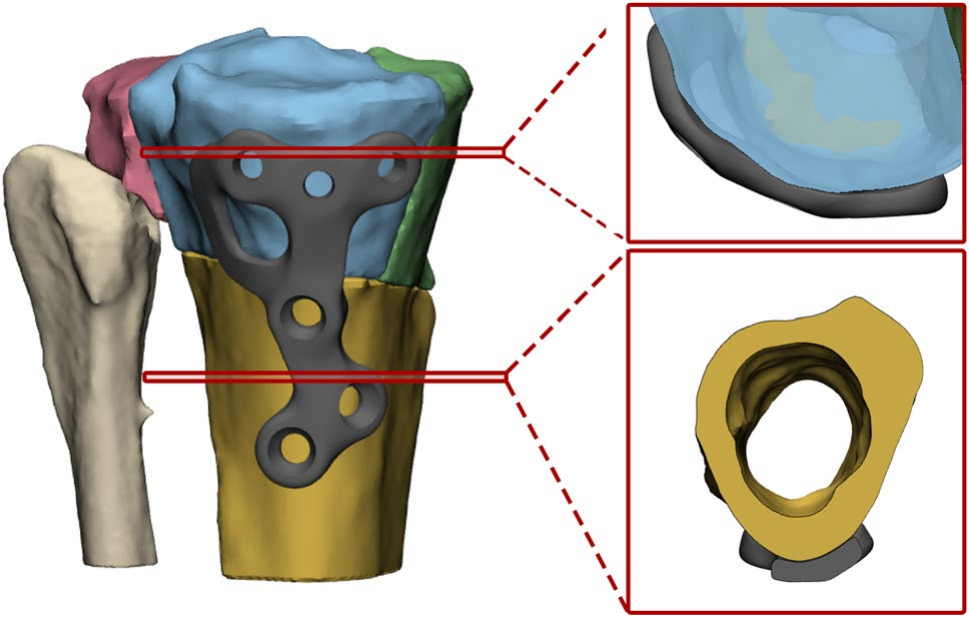
Unique features of the patient-specific implant (case 2, left): The distal part of the implant followed an S-shape which covers the margo medialis of the proximal tibia, whereas the proximal part of the implant was designed to follow the curvature of the medial condyl of the proximal tibia (right). This unique combination forces the tibial shaft and the fragments in the desired sagittal alignment

The patient-specific osteosynthesis plates were designed with 3-Matic software (version 17.0 Materialise), Solidworks Professional software (version 2020, Dassault Systèmes Solidworks), and the Geomagic package for Solidworks (3D Systems). The implant was designed to fit the specific anatomy such that the plate could serve as a template which facilitates the fracture reduction. Minimal clearance (± 0.2 mm) between the bone and implant was used to ensure a proper fit. For optimal fitting on the bone, the patient-specific implants were designed in such a way that the distal part of the implant followed an S-shape which covers the margo medialis of the proximal tibia (Fig. 3, bottom right). In addition, the proximal part of the implant was designed to follow the curvature of the medial tibial condyle of the proximal tibia just below the articular surface (Fig. 3, upper right). The unique features of the implant force the fracture fragments in correct alignment with the tibial shaft when applying compression. In addition, the fitting of the implant provides direct feedback to the surgeon regarding the fracture reduction, since poor fitting suggests that the fracture reduction in suboptimal.

**Fig. 4:**
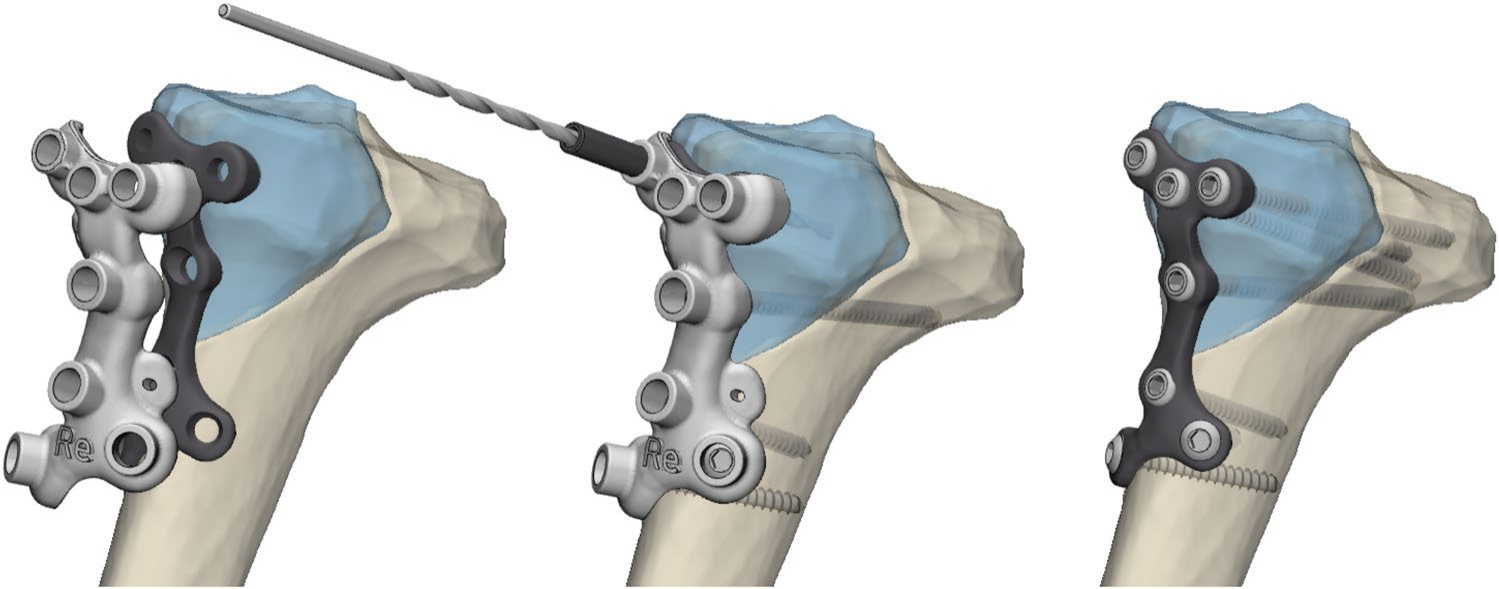
3D virtual surgical planning (case 3, right knee): *Left*) The implant and surgical guide, which were designed to fit a pure medial fracture of the proximal tibia. *Middle*) In the surgical guide, which fits on top of the designed implant, drill sleeves can be placed which direct the drill bit to the predetermined screw trajectories. The cylindrical holes within the surgical guides were designed to fit the drill sleeves. In addition the screwheads of the subsequently placed screws also fit through these cylinders so that it allows the screws to be placed without removal of the guide. *Right*) Final position of the patient-specific implant and screws

In this series, the proximal screws holes were designed to fit 3.5 mm locking screws, whereas the distal screw holes along the shaft of the tibia fit 3.5 mm cortical screw heads. For future application, choice for locking or cortical screws could be personalized based on the surgeons’ preference. The patient-specific plate is accompanied with a surgical guide, which fitted on top of the plate and facilitated the surgeon in positioning the plate and achieving the preplanned screw trajectories by drilling through the drill sleeve (Fig. 4).

### Surgical procedure

**Fig. 5:**
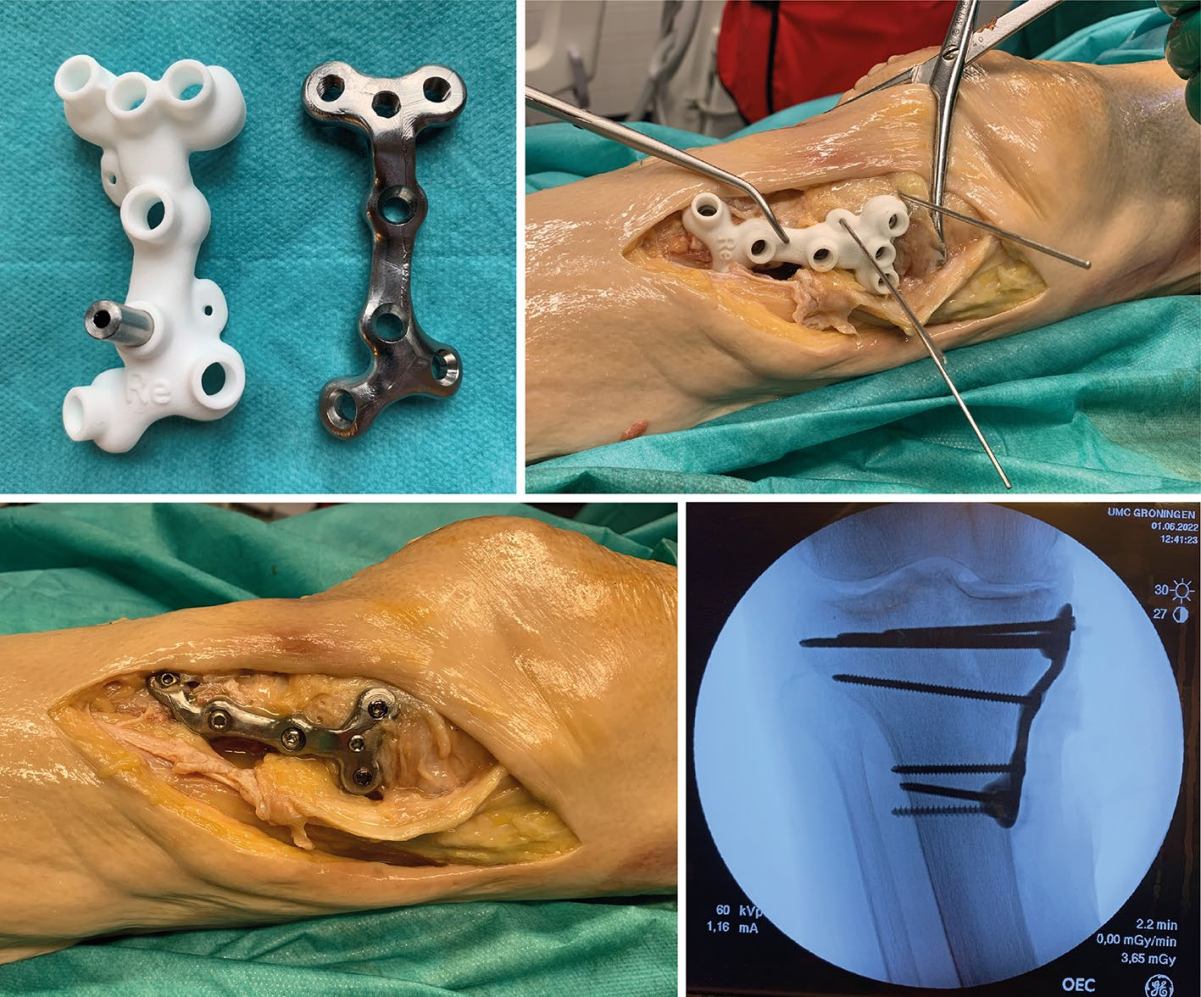
Surgical procedure of a pure medial split fracture (Schatzker 4). 1) After fabrication, the patient-specific implant and surgical guide can be sterilized and brought to the operating room (upper left). 2) Through a bilateral approach, the fracture was reduced and plate and guide were positioned after which the preplanned screw trajectories could be drilled (upper right). 3) The surgical guide was removed after placement of the screws (lower left). 4) The implant and screw positions were verified by fluoroscopy before wound closure (lower right)

**Fig. 6:**
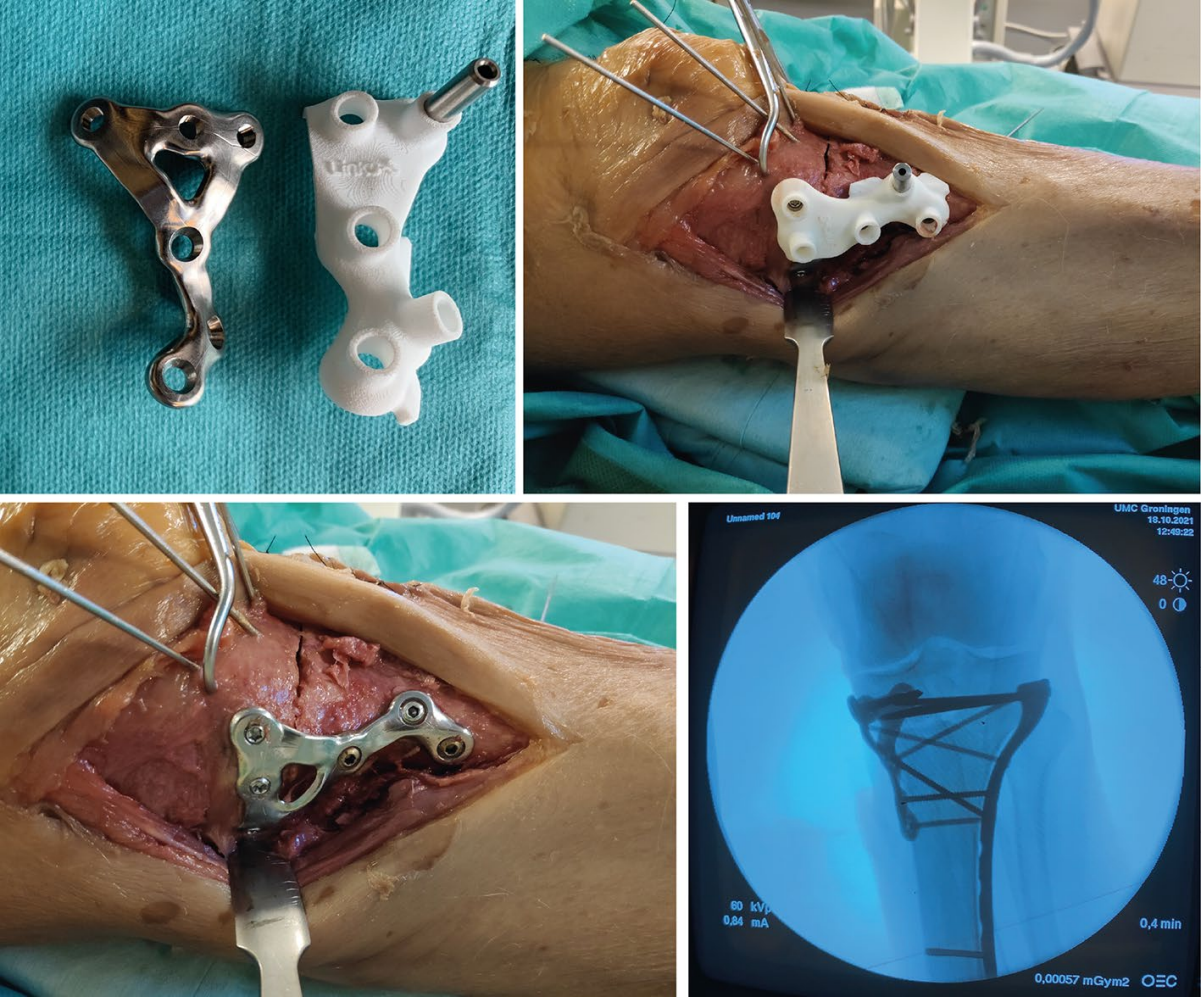
Surgical procedure of a full articular fracture (Schatzker 6). 1) After fabrication, the patient-specific implant and surgical guide can be sterilized and used during the operation (upper left). 2) Through a bilateral approach, the fracture was reduced and plate and guide were positioned after which the preplanned screw trajectories could be drilled (upper right). 3) The surgical guide was removed after insertion of the screws (lower left). 4) The implant and screw positions were recorded by fluoroscopy before wound closure (lower right)

The operations were performed by a consultant trauma surgeon. In three cadaver knees with an isolated medial fracture, a posteromedial surgical approach was performed with the patient in supine position. The posteromedial approach consisted of a longitudinal incision overlying the posteromedial border of the proximal tibia. The plane between the pes anserinus (anteriorly) and the medial head of the gastrocnemius (posteriorly) was developed by which the posteromedial border of the tibia was exposed. In three cadaver knees with a complete articular fracture, bilateral approaches (anterolateral and posteromedial) were performed. After reduction of the fractures, the patient-specific implants combined with the surgical guides were positioned according to the 3D surgical plan and verified using intraoperative fluoroscopy. A drill sleeve was inserted into the cylinder holes of the guide, and through this sleeve the screw trajectory was drilled. After drilling, the sleeve was removed and the screw was inserted while leaving the guide in place. After placing all screws, the guide was removed. In the specimens treated with a bilateral approach, a conventional lateral locking plate was then placed in addition to the patient-specific posteromedial implant. The implant and screw positions were verified by fluoroscopy before wound closure. Figure 5 and 6 depict the surgical procedure for both single and dual plating.

### Postoperative measurements

For each cadaver, a postoperative CT scan (0.6 mm slice thickness; iterative metal artefact reduction) was made to evaluate the articular reduction, tibial alignment, plate positioning and screw directions. The postoperative CT data was used to generate a 3D model of the reconstructed tibial plateau with plate and screws in situ.

#### Articular reduction and tibial alignment

Articular reduction was assessed by measuring the maximum residual gap and step-off on the CT slices. A gap was defined as a separation of fracture fragments along the articular surface. A step-off was characterized as a separation of fracture fragments perpendicular to the articular surface [15]. From the postoperative CT scan, pure anteroposterior and lateral radiographs were obtained using the Mimics software. Coronal alignment was then assessed by measuring the Medial Proximal Tibial Angle (MPTA) on the postoperative anteroposterior radiograph, whereas sagittal alignment was assessed by measuring the Posterior Proximal Tibial Angle (PPTA) on the postoperative lateral radiograph. The articular reduction was defined as adequate when both the residual gap and step-off were ≤ 2 mm, coronal alignment when the MPTA was 87 ± 5°, and sagittal alignment when the PPTA was 9 ± 5° [10, 16].

#### Plate positioning

**Fig. 7:**
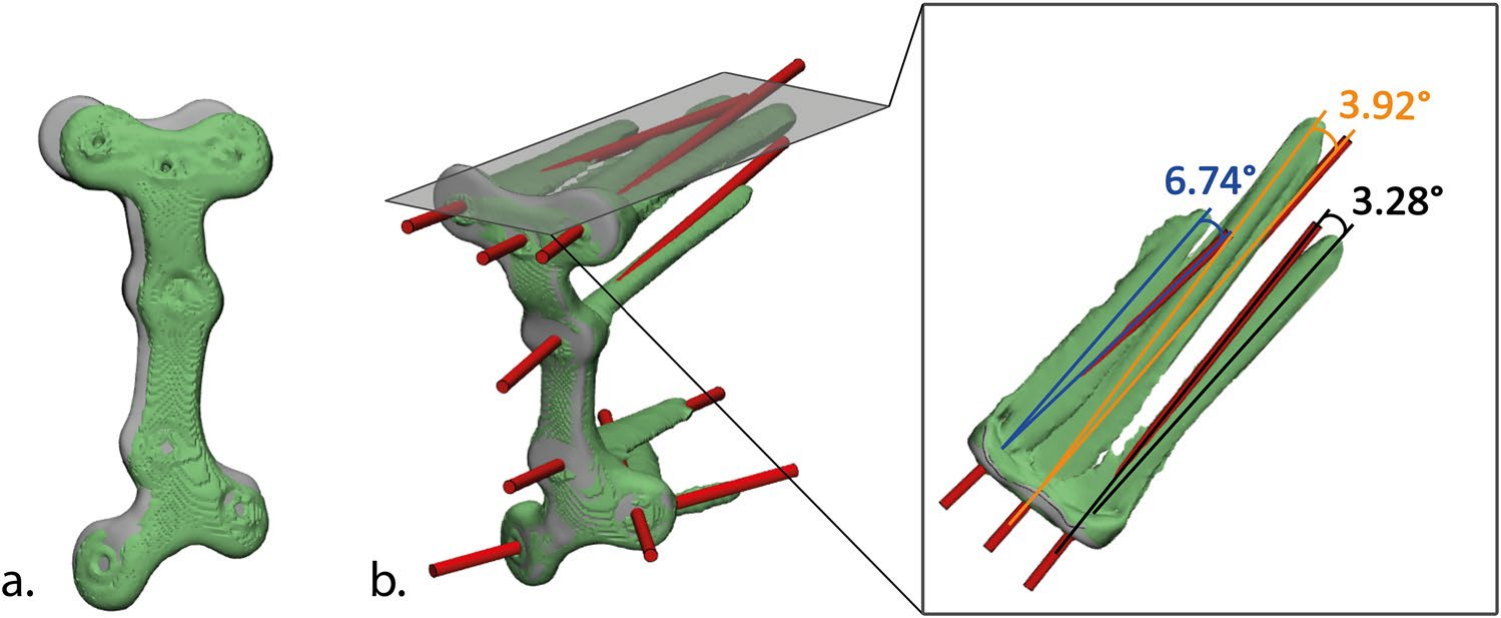
Postoperative evaluation: **a** The planned (gray) and achieved (green) position of the plate. Plate positioning was assessed by measuring the Euclidean distance between their center of gravities. **b** Measurement of the deviation between the achieved (green) and planned (red) screw directions (colour figure online)

We designed a plate that facilitates placement in a medial position (Fig. 5) and another plate that can be positioned further towards the posteromedial direction (Fig. 6). In order to compare the definitive positioning of the plate with the planned position, the 3D model of the postoperative tibial plateau was aligned with the 3D model of the surgical planning using the global registration function in 3-Matic version 15.0 (Materialise). The accuracy of positioning of the plate was obtained by measuring the Euclidean distance in millimeters between the center of gravity of the plate in the planned position and the plate in the postoperative position (Fig. 7a).

#### Screw directions and length

The lengths of the screws were determined in the preoperative surgical plan. Occurrence of screw penetration was considered when screws protrude the second cortex with more than 2 mm. The differences in screw direction were assessed by comparing the planned and postoperative screw trajectories, through matching of the postoperative plate (e.g., retrieved from the postoperative CT scan) with the planned plate (Fig. 7b). The 3D deviation in screw direction was measured between the inertia axes of the planned and postoperative screw trajectories in degrees.

## Results

A total of six tibial plateau fractures were operated using a patient-specific plate. Three fractures (right knees) consisted of an isolated medial fracture for which a medial patient-specific plate was designed and implanted. The other three fractures (left knees) consisted of multifragmentary complete articular fractures and were treated with dual plating: A conventional lateral locking plate (VA-LCP, DePuy Synthes) in combination with a patient-specific posteromedial plate. All articular reductions were considered adequate (gaps and step-offs < 2 mm), except in one case where the step-off was slightly higher with 2.8 mm (case 2, right knee). In terms of tibial alignment, all cases showed an adequate postoperative coronal (87 ± 5°) and sagittal (9 ± 5°) alignment. Table 1 describes the initial and postoperative articular incongruencies as well as the postoperative alignment for each case.

**Table 1:** Articular reduction in terms of gap (mm) and step-off (mm), and postoperative tibial alignment(**°**)

Case	Fracture classification	Gap (mm)		Step-off (mm)		Alignment (°)	
		Preoperative	Postoperative	Preoperative	Postoperative	MPTA	PPTA
Case 1–Left knee	Schatzker 6	11.5	1	5.5	1.2	88.5	10.0
Case 1–Right knee	Schatzker 4	7.4	1.9	2.8	0.8	88.0	11.5
Case 2–Left knee	Schatzker 6	4.6	1.5	4.8	1.5	87.5	6.5
Case 2–Right knee	Schatzker 4	10.2	0	5.5	2.8	85.5	8.0
Case 3–Left knee	Schatzker 6	4.2	0.8	4	1.4	86.0	8.5
Case 3–Right knee	Schatzker 4	4.5	0	4.7	0	86.0	10.0
Median (IQR)		6.0 (4.5–9.5)	0.9 (0.2–1.4)	4.8 (4.1–5.3)	1.3 (0.9–1.5)	86.8 (86.0–87.9)	9.3 (8.1–10.0)

The six placed patient-specific plates had a median deviation of 3.0 mm (IQR: 2.8–3.7 mm) from their positions in the 3D surgical planning. A total of 41 screws were placed using the drilling guides. The placed screws showed a median deviation of 3.3° (IQR: 2.5–5.1°) when compared with the planned direction of the screws (Table 2). No screw penetration was observed.

**Table 2:** Difference in position of the plate (mm) and the deviation in screw directions (°) between the planned and the achieved position of plate and screws

Case	Fracture classification	Position plate	Direction screws	
		∆ Distance (mm)	N	∆ Direction (°)
Case 1–Left knee	Schatzker 6	2.9	5	3.1 (1.7)
Case 1–Right knee	Schatzker 4	3.9	8	3.3 (1.3)
Case 2–Left knee	Schatzker 6	4.5	6	2.7 (0.6)
Case 2–Right knee	Schatzker 4	2.8	7	6.1 (3.7)
Case 3–Left knee	Schatzker 6	3.1	8	3.9 (2.2)
Case 3–Right knee	Schatzker 4	2.8	7	4.1 (3.5)
Median (IQR)		3.0 (2.8–3.7)	7 (6.3–7.8)	3.3 (2.5–5.1)

## Discussion

This case series demonstrates that 3D virtual surgical planning including patient-specific osteosynthesis combined with drilling guides is feasible in the surgical treatment of tibial plateau fractures with involvement of the medial plateau. The application of patient-specific osteosynthesis plates with drilling guides not only facilitates accurate plate and screw positions according to the preoperative plan, but also allows for adequate surgical reduction.

In the last years, 3D-assisted surgery emerged in the treatment of tibial plateau fractures. This includes a spectrum of modalities, such as 3D printing models, pre-contouring of osteosynthesis material and surgical guides [17]. Patient-specific implants have also found their way into clinical practice in the field of acetabular fracture surgery [11, 12]. To our knowledge, no patient-specific plate has been used in the clinical treatment of tibial plateau fractures, though some concept implants have been introduced. Teo et al. recently showed the feasibility of 3D-printed patient-specific locking plates for lateral tibial plateau fractures [18, 19]. However, they focused more on the feasibility and time management of the production and the biomechanical strength of the construct, rather than the surgical advantages. Also, for treatment of Schatzker 2 fractures conventional implants may suffice. Our proposed technique is assumed to be of additional value for complex fractures or tibial bones that differ from the mean tibial shape and could therefore benefit from the personalized shape of the implant. Schmutz et al. state that conventional implants were designed with the view to fit the 50^th^ percentile of the population [20]. Therefore, especially tibial anatomy that differ from the mean shape (*e.g.,* pre-existent bone deformities, the previous fractures, or comminuted acute fractures) are assumed to benefit from personalized techniques.

The surgical treatment with the use of a patient-specific implant, led to a satisfactory tibial alignment in all treated fractures and in five (out of six) to adequate surgical reduction. One case showed a postoperative step-off of 2.8 mm, which was slightly higher than the generally accepted 2 mm cut-off. In addition, the use of the custom-made patient-specific implants was considered easy to handle (e.g., positioning and fitting according to virtual planning) by the operating surgeon. The rationale behind a custom-made patient-specific implant is that it can serve as a template which facilitates the fracture reduction and restoring tibial alignment. This study shows promising results regarding the latter. In this study, we used a posteromedial approach with the body placed in a supine position. We presented a plate that can be placed in a medial position (Fig. 5) and another plate that can be placed posteromedial (Fig. 6).In clinical practice, the preferred plate positioning will obviously depend on fracture morphology. However, it should be acknowledged that positioning of the patient and exposure of the medial tibial condyle may differ between surgeons, which might limit the use of a plate or guide as a fracture reduction device. A future study should validate these results in a clinical setting. Applying these tibial implants in clinical practice should be feasible, because patient-specific plates have already been designed, produced, and clinically applied in different body regions in our clinic [11, 12].

The placement of the screws through the surgical guides went without any difficulties. Only one screw could not be placed through the guide due to blocking by the soft tissue, but could be placed after removal of the surgical guide. Huang et al. recently used patient-specific surgical guides in combination with conventional implants in tibial plateau fracture surgery, which resulted in an average difference between planned and achieved screw trajectory of 6.3 ± 3.4° [21]. In addition, our research group recently assessed the use of surgical guides in the treatment of acetabular fractures. In this cadaver study, median difference between planned and achieved screw trajectory was 5.9° (IQR: 4–8°) [22]. Both these studies, however, used these surgical guides in combination with conventional implants. This current study showed superior results with a median difference between planned and achieved screw trajectories of 3.3° (IQR: 2.6°), which may be facilitated by the use of a patient-specific implant.

One of the limitations of this study is that it is an experimental design with a limited number of human cadavers. The fractures were created with the use of an oscillating saw in combination with an osteotome, which were similar but not identical to fractures seen in clinical practice. The use of the osteotome introduced some plastic deformation and removal of bone, especially along the corners of the fragments. This complicated the anatomical reduction of the fragments. Moreover, no control group was used to compare fracture fixation with a patient-specific plate to a conventional plate. The reason for this is that it is already clear from clinical practice that currently available medial tibial plateau plates often do not fit properly. This study only aimed to assess the feasibility of patient-specific plates for medial tibial plateau fractures. Future challenges are related to manufacturing time and applicability of the workflow. Previous clinical studies and clinical experience, however, show that these implants can be designed and manufactured within four days, fitting within the clinical timeline for treatment of tibial plateau fractures [11]. However, we realize that this innovative workflow requires substantial resources, including a dedicated team, validated software packages and an osteosynthesis plate production facility. The associated costs for these resources were not part of this feasibility study. Also, only two different types of fracture patterns were included in this study and treated by one experienced surgeon. A next step would be to perform a clinical study to assess surgical parameters, patient-reported outcomes, and cost-effectiveness in order to determine which medial tibial plateau fracture patterns would benefit most from fixation with a patient-specific plate.

In conclusion, this feasibility study described the development and implementation of 3D virtual surgical planning including patient-specific osteosynthesis for medial tibial plateau fracture surgery. This study showed that the use of custom-made osteosynthesis plates facilitates proper fracture reduction and tibial alignment. Moreover, all screws could be placed according to accurately using the accompanied drilling guides.

## Data Availability

Not applicable.
